# Dual-view weakly-supervised learning for apple tree flower counting

**DOI:** 10.3389/fpls.2026.1869941

**Published:** 2026-07-09

**Authors:** Martin Vitousek, Jan Vrba, Matous Cejnek, Jakub Jura, Klara Schankova

**Affiliations:** 1 Department of Instrumentation and Control Engineering, Faculty of Mechanical Engineering, Czech Technical University in Prague, Prague, Czechia; 2 Research and Breeding Institute of Pomology Holovousy Ltd., Holovousy, Czechia

**Keywords:** apple tree, deep learning, machine vision, precision agriculture, weakly-supervised learning

## Abstract

This paper presents a method to estimate apple tree flower cluster count using image analysis techniques. The main research question is how accurately flower clusters on apple trees can be counted using a camera-based approach and weakly-supervised learning, compared to traditional visual estimation. A camera is used to capture images of blooming apple trees from two sides. These images are processed by a weakly-supervised model based on a ResNet feature extractor and a feature pyramid network serving as the feature aggregator. The model is trained and validated using reference data obtained through manual flower cluster counts in the orchard and from estimations based on camera images. The model was trained using field-validated data and visually-estimated data, enabling a comparative evaluation. The proposed model surpassed conventional image segmentation methods in estimating both manually counted and visually estimated flower clusters. The proposed method achieves a relative error of 10.26% in estimating flower cluster quantities, demonstrating its effectiveness and improved accuracy over traditional approaches. Its reliance on field-validated reference data adds to its robustness and practical relevance.

## 1 Introduction

Flower count plays a crucial role in predicting yield and managing crop load in apple orchard management (Verma et al., 2023). An excessive number of flowers can result in suboptimal apple quality and orchard overcropping (Wertheim and Webster, 2005) or biennial bearing (Campbell and Kalcsits, 2024), while accurate flower set count prediction can effectively and precisely define the thinning management, and thus significantly reduce costs associated with labor, resource allocation, and overall management (Robinson et al., 2013; Bound, 2018).

However, accurately counting flowers manually presents a formidable challenge. This process is time-consuming, labor-intensive, and prone to errors. For large orchards, it is virtually impossible to count all flowers on all trees within a short time span, further emphasizing the need for a scalable and efficient alternative (Robinson et al., 2022). Addressing this challenge, this study investigates automated flower cluster counting, aiming to improve orchard management practices with a more reliable and time-efficient approach.

Gradually increasing computational power allowed machine vision to establish itself in precision agriculture (Tian et al., 2020) and in orchard yield prediction (He et al., 2022). Advances in computational power and a growing number of various data sets enabled the development of deep learning models, especially focused on segmentation and object detection, that have dominated RGB image processing since 2012 (Zou et al., 2023). These models found their application in orchard pruning (Zahid et al., 2021), harvesting (Altaheri et al., 2019), and even pest monitoring (Chen et al., 2021).

Deep learning techniques depend greatly on high-quality annotations for training precise models. When it comes to counting flowers, this requirement poses considerable challenges. Flowers may appear in different phenological stages with variations in size, shape, and color, complicating consistent and accurate annotation. Annotating trees with a high density of flowers is especially demanding, particularly when using detailed polygon annotations to delineate individual flowers or clusters, which can lead to errors owing to human fatigue or image ambiguity.

Estimating the overall number of flower sets on a tree from one image presents a significant challenge. A photograph usually shows just a fraction of the tree, and the flower distribution can vary greatly across various sections, which can result in either underestimating or overestimating the total number. Additionally, occlusion from overlapping flowers, leaves, or branches makes the annotation and counting process more complex, affecting the model’s accuracy and reliability.

Challenges related to annotation in deep learning can be alleviated by weakly supervised learning. Rather than depending on intricate annotations like exact positions and outlines of flower clusters, weakly supervised strategies employ more straightforward labels, such as the overall count of flower clusters within the image. This method lessens reliance on labor-intensive and error-prone annotation procedures and has been investigated in tasks involving flower cluster count estimation.

Weakly supervised methods help reduce the effort needed for annotation, yet they still have limitations. Existing techniques mainly concentrate on estimating the flower cluster count in single images, neglecting the challenge of combining counts from multiple images of the same tree. This gap frequently leads to overcounting and hinders precise estimation of a tree’s total flower or flower cluster count, which is essential for effective orchard management. Therefore, although weakly supervised learning presents a promising avenue, advancements are essential to address these shortcomings and achieve dependable flower counting at the tree level.

In our study, we merge the ideas of weekly-supervised learning and multiview approach in order to effectively overcome the challenges posed by the complex nature of data annotations, truth representation, and partial information. The main contributions of the article are as follows:

- Evaluate and quantify the challenges of real-world flower cluster counting by leveraging actual physical flower cluster counts instead of relying solely on image annotations, addressing the limitations of annotation-based methods.
- Introduce a dual-view weakly supervised regression framework that estimates whole-tree flower cluster counts directly from field-validated labels, eliminating the need for labor-intensive object-level annotations. Unlike existing approaches that operate on single images and estimate only visible flowers, the proposed method aggregates complementary information from opposite sides of the tree to improve estimation of the actual flower cluster count.

## 2 Related works

Recent studies on flower count estimation address the problem from two distinct perspectives: object detection-based counting and weakly supervised regression approaches.

### 2.1 Object detection-based counting

There are already applications of deep learning models for flower set counting. In the study (Farjon et al., 2020) the authors use the Faster R-CNN architecture with VGG16 backbone (Simonyan and Zisserman, 2014), which was pre-trained in the ImageNet dataset (Deng et al., 2009). They aim to estimate the flowering intensity and determine the blooming peak date to enable better chemical thinning decisions. They achieved an average precision score of 0.683 for flower detection.

In (Dias et al., 2018) authors proposed the convolutional neural network (CNN) with the last layer replaced by a support vector machine (SVM) classifier to detect apple flowers. The proposed CNN works as a feature extractor for regions obtained by a simple linear iterative clustering superpixel algorithm (Achanta et al., 2012). After applying PCA to those features, the SVM performs the final classification of the proposed region. This approach results in recall and precision rates that are slightly higher than 90%.

In the study (Mann et al., 2022) a flower detection model based on Mask R-CNN (He et al., 2017) is presented. The authors downscaled the original images with 6080x3420 resolution to 760x427 to achieve recall 90.7% and precision 91.8% Another application of Mask R-CNN is presented in (Mu et al., 2023), in order to detect apple flower and identify the king flower during multiple flowering stages. The authors distinguish between exposed flowers, occluded flowers, and flower buds, and present the overall precision 78.1% with the recall rate 71.3%. They concluded that overall performance did not meet the commercialization requirement.

In the study (Li et al., 2022) the successful deployment of the YOLOv3 (Redmon and Farhadi, 2018) and YOLOv4 (Bochkovskiy et al., 2020) models for the detection of kiwifruit flowers and buds is presented. They achieve 95.2% mAP with YOLOv3 and 97.6% mAP with YOLOv4 while maintaining high precision (98% for YOLOv3 and 97% for YOLOv4) and recall (95% for YOLOv3 and 96% for YOLOv4).

In (Palacios et al., 2020) a grapevine flower detection and quantification problem is approached. The authors use the SegNet architecture (Badrinarayanan et al., 2017) with the VGG19 network as encoder, resulting in the 73% F1 score. To obtain a real number of flowers, they proposed a linear regression model that achieved *R*^2^ from 0.73 to 0.96 depending on the variety.

The problem of flower detection and counting in highly populated images is addressed in (Estrada et al., 2024). The authors process images with resolution 1280x720 that have more than 400 flowers on average. They employed YOLOv5 (Jocher, 2020), YOLOv7 (Wang et al., 2023) and YOLOv8 (Jocher et al., 2023) object detection models and a multicolumn deep neural network (Ciregan et al., 2012) for density map prediction. The average error of the predicted density map was 9.98%, while the best YOLOv7x model achieved 29.7%.

The estimation of the number of litchi flowers is approached in (Lin et al., 2022) via a multicolumn neural network to predict the density map. The litchi flowers are specific due to their small size but large quantities. The authors propose the use of linear regression on the obtained density map and achieve a mean absolute error of 16.3 flowers per image.

A common practice in the presented studies is the use of transfer learning and pre-training models in the COCO data set (Lin et al., 2015) or ImageNet (Deng et al., 2009). This approach seems feasible, as it in many cases improved the quality of the models significantly. Another commonly used method is data augmentation (Shorten and Khoshgoftaar, 2019) as the examined datasets contain only a limited number of samples. Augmentation methods are often vertical flipping, horizontal flipping, brightness transformation, and rotation.

As observed, the scientific community has already put considerable effort into counting flowers. Nonetheless, it is crucial to mention that the detection and segmentation methods employed in the cited studies do not account for the actual flower count but rather the count of flowers visible in images. Recent research (Tan et al., 2024; Zheng et al., 2023) has attempted to tackle this limitation by employing a multiview strategy. This technique involves compiling data from multiple images, thereby reducing errors associated with obscured flower clusters.

Recent counting research has increasingly explored transformer-based architectures. For example, CountFormer (Mo et al., 2024) employs transformer modules to aggregate information across multiple views for crowd counting, while more recent approaches continue to improve transformer-based counting performance through increasingly sophisticated multimodal designs (Meng et al., 2025). Although these methods demonstrate strong performance on large-scale counting benchmarks, they generally require substantially larger datasets, dense annotations, and involve considerably higher model complexity. Such requirements are difficult to satisfy in precision agriculture, where obtaining large annotated datasets is expensive and time-consuming. Furthermore, transformer architectures often contain a large number of trainable parameters, increasing the risk of overfitting when only limited agricultural data are available. Consequently, we focus on convolutional regression-based approaches that better align with the practical constraints of agricultural applications, including limited data availability, reduced annotation effort, and the need for robust generalization.

### 2.2 Weakly supervised-based counting

Weakly supervised counting offers a simplified approach, from the annotation effort point of view, to estimating the number of flowers that are hard to see by completely sidestepping complex data annotation. This can be achieved through techniques such as weakly-supervised learning or feature-based regression (Seguí et al., 2015). Although this method appears to be a more intuitively sound solution for flower cluster counting, there is a shortage of supporting studies in this field. A comparative study of deep regression and detection-based counting approaches on multiple agricultural datasets was performed in (Gomez et al., 2021). According to the results, the counting-by-regression outperformed counting-by-detection at high object densities, while giving comparable performance at lower densities.

In the study (Bhattarai and Karkee, 2022), the authors present a weakly supervised network CountNet, that reached 18.4 root mean square error on the studied dataset. However, the evaluation is against single-image visual estimations of flower count, which may not directly correspond to the actual flower count on a tree. Their work is furthered in the research (Bhattarai et al., 2024), introducing AgRegNet, a deep regression network features integrated segmentation and attention mechanisms specifically for flower and fruit density in orchards. The authors utilize point annotation data, where each object is represented by a single pixel located in the center of a flower or apple. AgRegNet achieved a root mean square error of 23.8 in flower count estimation for trees within varying orchard configurations, such as fruit wall and V-trellis structures. While density-regression ideas were partly inspired by the crowd-counting literature, recent work in precision horticulture has increasingly shifted toward orchard-specific models that explicitly address canopy geometry, variable illumination, and severe occlusion. Representative examples include tree-level apple inflorescence detection and density mapping with MTYOLOX (Xia et al., 2023), apple counting pipelines that combine object detection with multi-object tracking in orchard videos (Hu et al., 2023; Yang et al., 2024), point-supervised regression for joint flower and fruit density estimation, localization, and counting in orchards (Bhattarai et al., 2024), UAV-based tree-level apple flower quantification (Shang et al., 2024), multitask regression for single-panicle litchi flower counting (Lin et al., 2024), and lightweight apple flower detectors designed for natural orchard environments (Wang et al., 2025). Compared with generic crowd-counting models, these studies are better aligned with precision-agriculture applications because they are developed for fruit-tree scenes and emphasize robustness to clustered targets, foliage occlusion, and practical field acquisition conditions. Nevertheless, these methods typically rely on some form of object-level supervision, such as bounding boxes, point annotations, segmentation masks, density maps, or object tracking labels. In contrast, the proposed method is trained solely from tree-level flower cluster counts and does not require any annotation of individual flower clusters or their locations.

Recent weakly supervised counting approaches have increasingly incorporated transformer architectures to improve global context modeling. For example, Cai et al. (Cai et al., 2024) combined CNNs and transformers for weakly supervised counting, while Mao et al. (Mao et al., 2025) proposed a feature-adaptive fusion framework under limited supervision. Despite their promising performance, these methods generally require larger datasets and more detailed annotations than are typically available in agricultural applications. In contrast, our approach focuses on practical orchard scenarios characterized by limited training data, minimal annotation effort, and the need to integrate information from multiple viewpoints to estimate whole-tree flower cluster counts.

## 3 Materials and methods

This section details the proposed method along with the data utilized. The dataset and the subject of data annotation are discussed initially, as the various annotation techniques are essential to this research.

### 3.1 Dataset

This study utilizes a unique dataset of images depicting flowering trees, with a few exceptions of non-flowering trees. The dataset consists of 73 columnar apple trees, categorized by cultivars as follows: 33 Redspring, 20 Goldlane, and 20 Rumba. The orchard used for data collection is located in a low-populated rural area (50.3712739N, 15.5693422E) in Czechia. The images in the used dataset were obtained using Basler camera ace acA4600-7gc with 3.5 mm lens (F/2mm, f/1.8) with resolution 4608x3288px. Images were cropped manually to a custom resolution to feature only the tree crown. The varying resolution of differently cropped images was rescaled to 512x512 px. Images were taken from orchards during the years 2022 and 2023. The camera–tree distance was in the range of 100–120 cm. This distance represents half or less of the width of the orchard path. In practical applications, a shorter distance would require a much larger field of view, and a greater distance is not possible due to space restrictions. The light conditions vary greatly among the images: good visibility during the day, very poor visibility during the late evening (lower tens of W/m² of solar radiation), and artificial lighting during the night (2x35 W LED light). To summarize the high diversity of the images in our dataset, the images contain different tree cultivars, trees of different ages, and various light conditions. In terms of flowering stage (BBCH scale), flower clusters were counted from BBCH 57 (pink bud) to BBCH 65 (full flowering) (Meier et al., 2009; Rea and Eccel, 2006).

The dataset includes 73 distinct trees, with each tree having one or more observations. An observation consists of a pair of images taken from opposite sides at nearly the same time. Observations were made both during daytime and nighttime, and for some trees, both night and day observations are available. In total, there are 117 observations (image pairs).

Three data labels are used: flower cluster count obtained in the field (field-validated), flower cluster count visual estimation (visually-estimated), and polygon annotations of flower clusters in images. The field-validated count is the most accurate information obtained by experts who combine visual inspection with physical verification through tactile assessment, using their hands or fingers to confirm the presence of individual flower clusters during counting. It is labor-intensive and time-consuming to acquire such information, and therefore it is rarely used in studies. It is also unsuitable for typical object detection methods that assume all flower clusters are visible in the images taken.

Expert operators also employ visually-estimated count as another metric. The process involves the operator viewing two images of a tree and counting the flower clusters, using common sense to prevent counting the same flower cluster twice across both images. This procedure is repeated three times, with the median count recorded. Discrepancies from the field-validated counts may arise due to factors such as poor camera angles and the time gap between collecting the images and establishing the field-validated counts.

The final type of annotation consists of polygon delineations of flower clusters within images. Although these annotations are not used for training the proposed weakly supervised regression model, they are included for two reasons: (i) to enable comparison with conventional detection-based approaches such as YOLO, and (ii) to analyze the limitations of annotation-driven counting, including double counting, fragmentation, and occlusion effects.

**Figure 1:**
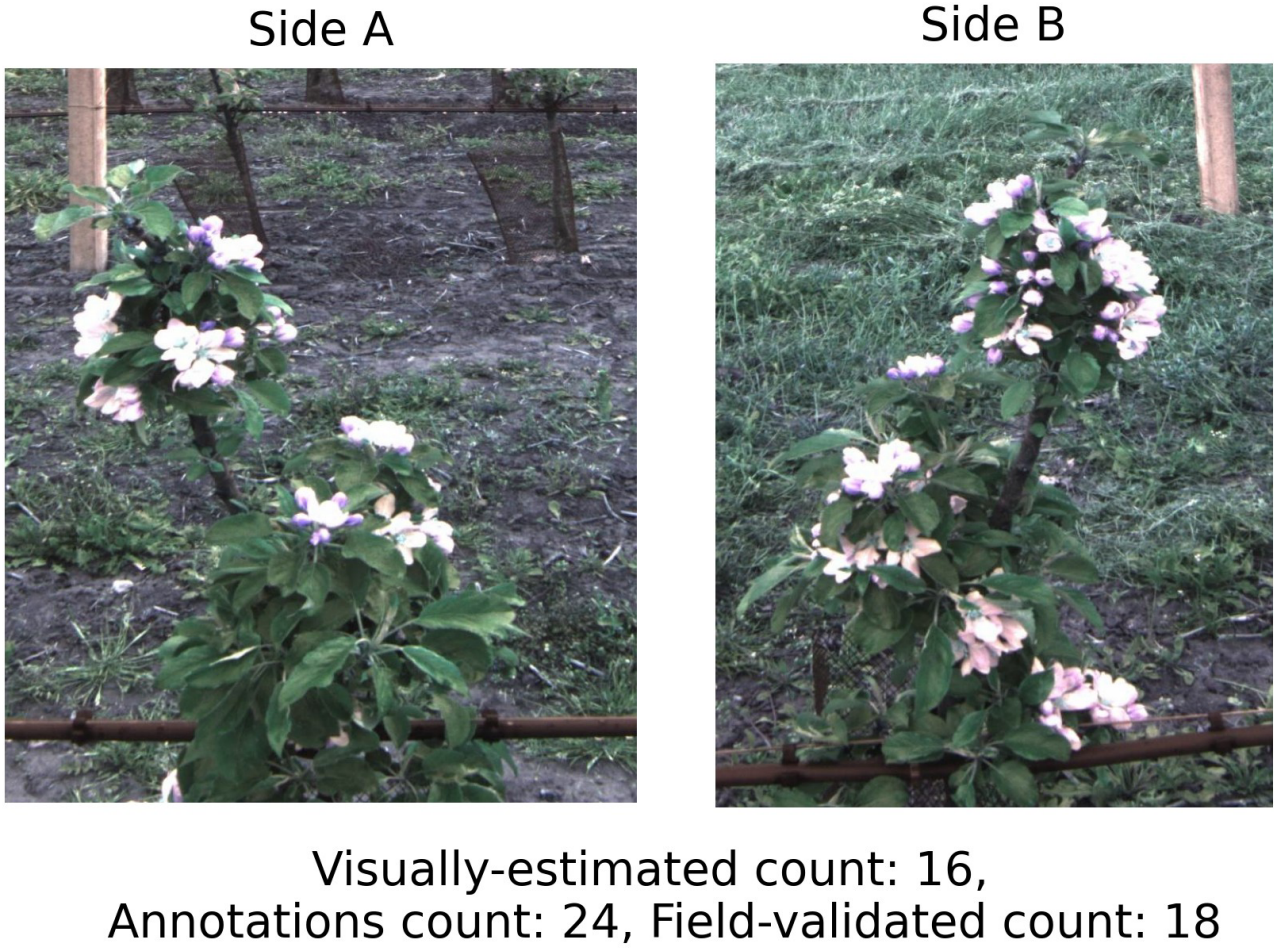
Example of different annotated trees in different light conditions. The upper row shows images acquired during daylight, while the lower row shows images acquired at night using artificial lighting.

The practical properties of the different label sets are summarized in **Table 1**. **Table 2** illustrates the distinctions among the three labeling techniques employed in this study. Polygon annotations, mainly used for object detection, demonstrate the highest mean, the broadest range, and the most variability, indicating potential issues such as double counting, inaccurate detection of fragments, and related errors. Conversely, visually-estimated counts may even tend to undercount, possibly because some flower clusters might not appear in the images. **Table 3** shows a comparison of flower cluster count data labels: mean difference and maximum difference are computed relative to the field-validated counts or visually-estimated counts. Pearson correlation coefficients reflect the measure of the linear relationship between the sources. Graphical examples of concrete trees and their labels are presented in **Figure 1**.

**Table 1:** Comparison of flower cluster count data descriptors across different metrics.

Metric	Field-validated	Visual-estimated	Polygon annotations
Data object	Tree	Image pair	Image
Data format	Scalar value	Scalar value	Polygon coordinates
Dual counting error	No	Limited	Yes
Fragmentation error	No	Limited	Yes
Occlusion error	No	Limited	Yes
Flower cluster location	Unknown	Unknown	Known

**Table 2:** Statistical properties of flower cluster counts obtained by different types of labeling: mean value, standard deviation, minimum and maximum value.

Count Source	Mean	Std	Min	Max
Field-validated Count	20.31	14.76	0	61
Visually-estimated Count	17.91	12.52	0	56
Polygon Annotations	24.66	16.26	0	75

**Table 3:** Comparison of flower cluster count data labels: mean difference, maximum difference and Pearson correlation.

Count source 1	Count source 2	Mean diff.	Max diff.	Correlation
Visually-estimated	Field-validated	2.71 (15.13%)	23	0.97
Polygon Annotations	Field-validated	5.75 (23.33%)	18	0.93
Polygon Annotations	Visually-estimated	7.28 (29.53%)	24	0.96

### 3.2 Proposed method

The proposed method utilizes weakly supervised learning by processing a pair of images corresponding to opposite sides of the same tree. Each image *x_n_*, *_o_*, *_s_* represents side s∈{A,B}, tree *n*, and observation *o*.

Both images are processed independently using a shared feature extraction network, producing feature vectors (Equation 1)

(1) $$h_{n,o,A}=f_{\theta }\left(x_{n,o,A}\right),\;h_{n,o,B}=f_{\theta }\left(x_{n,o,B}\right).$$

The resulting feature vectors are concatenated (Equation 2)

(2) $$h_{n,o}=\left[h_{n,o,A};h_{n,o,B}\right],$$

and fed into a regression head that predicts the whole-tree flower cluster count (Equation 3)

(3) $$y_{n,o}=g_{\phi }\left(h_{n,o}\right).$$

The training target *t_n_*, *_o_* corresponds either to the field-validated flower cluster count or the visually-estimated count.

This formulation represents a key distinction from existing weakly supervised counting approaches, which typically operate on single images and estimate only visible flowers. By jointly optimizing predictions from opposite sides of the same tree using field-validated whole-tree counts, the proposed framework learns to integrate complementary information from multiple viewpoints without requiring explicit correspondence matching or object-level annotations.

The AdamW (Loshchilov and Hutter, 2017) optimizer is employed for training, utilizing learning rate decay following a cosine annealing schedule, that repeats every 30 epochs. The initial learning rate is set to 10^−3^ and decreases to 10^−6^. Training was performed for 300 epochs, as extending the training further did not result in a significant improvement in validation performance. The SmoothL1Loss is used for loss calculations as follows (Equation 4):

(4) $$\text{SL}1\left(y_{n,o},t_{n,o}\right)=\{\begin{array}{ll} \frac{1}{2}{\left(y_{n,o}-t_{n,o}\right)}^{2} & \text{if }\left|y_{n,o}-t_{n,o}\right|<\beta ,\\ \left|y_{n,o}-t_{n,o}\right|-\frac{\beta }{2} & \text{otherwise} \end{array}$$

where *y_n_*, *_o_* is the regression output, *t_n_*, *_o_* is the target value (field-validated count or visually-estimated count), *β* = 1 is a positive constant that defines the boundary between the quadratic (L2) and linear (L1) regions. The value *β* = 1 was selected to match the unit scale of the target variable, providing a balance between sensitivity to small errors and robustness to larger counting deviations.

#### 3.2.1 Feature extraction

The proposed model uses Residual Network (ResNet) (He et al., 2015) with convolutional layers as a backbone. In ResNet, the main idea is the use of residual blocks. Each block has a shortcut connection that bypasses one or more layers, making it easier for the network to learn identity mappings (outputs that are identical to inputs for certain layers), if needed, rather than forcing every layer to transform the data in a complex way. This approach has made ResNet very effective and widely used in computer vision tasks. The concrete variant of ResNet used in this study is the PyTorch ResNet50 implementation pre-trained with the ImageNet dataset (Deng et al., 2009).

#### 3.2.2 Feature aggregation

**Figure 2:**
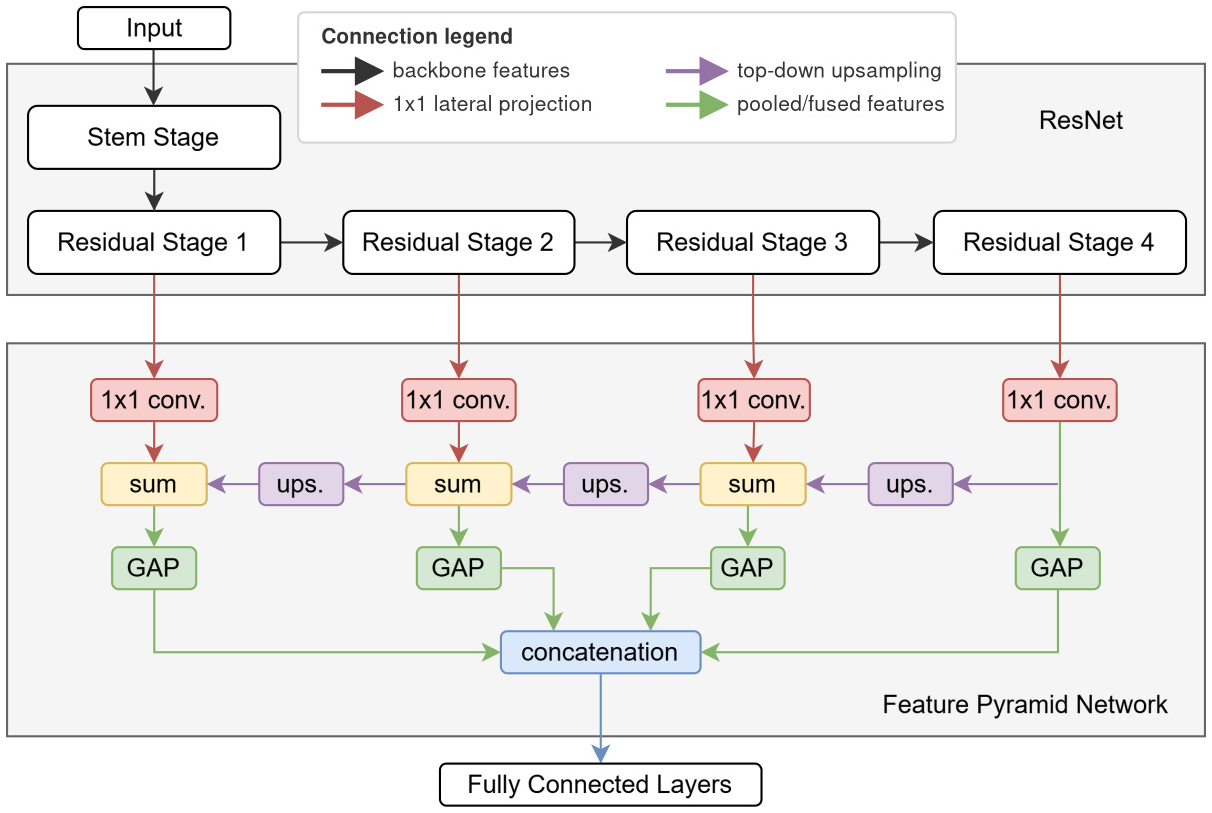
Architecture overview of the proposed dual-view framework. Images from both sides of the same tree are processed independently using shared network weights. Feature representations extracted from both views are concatenated and processed by a regression head to obtain the final whole-tree flower cluster prediction.

We propose the Feature Pyramid Network (FPN) (Lin et al., 2017) use in this model to perform feature aggregation by combining bottom-up and top-down pathways to enhance multiscale feature representations extracted from the ResNet backbone. This should be beneficial because of the varying sizes of the flower clusters and their fragments. Starting with the highest-level feature map, the deepest feature representation is reduced to a fixed number of channels through a convolutional operation and serves as the initial input to the top-down pathway. This feature map is then spatially upsampled by a factor of two and combined with a lateral feature map from a shallower layer that has been processed through another convolutional operation. This fusion generates the first intermediate feature representation. The process is iteratively repeated, where the intermediate feature maps are upsampled and fused with corresponding lateral features from progressively shallower layers, ultimately producing multiple output feature maps at different resolutions. The use of lateral connections and top-down upsampling ensures that the resulting features at each resolution are both semantically enriched and spatially detailed. Finally, the output feature maps from all scales are processed using global average pooling (GAP), and the resulting features are concatenated to form a unified representation for subsequent tasks. This hierarchical aggregation effectively integrates low-level spatial details with high-level semantic information, making the model well-suited for multiscale representation learning. The model architecture overview with a focus on the FPN section is shown in **Figure 2**. By iteratively upsampling and fusing features from different levels, the model creates a hierarchically enriched feature representation that benefits both small and large objects.

#### 3.2.3 Flower cluster count regression

The feature vector provided by the FPN is fed to the fully connected network designed for regression purposes. This network contains a sequence of three dense layers. The input features are reduced through progressively smaller layers: from input size to 512, to 256, and finally to a single output. Each layer applies the ReLU activation. The dropout for regularization is used after the first layer (20%) and the second layer (10%). The final layer outputs a single value with ReLU activation, satisfying the non-negative prediction requirement.

### 3.3 Reference methods

Two methods serve as our reference points. The first one is CountNet, selected due to its close resemblance to our approach. Further insights into CountNet can be found in subsection 3.3.1. The second reference method is YOLO, chosen for its widespread application in detection and segmentation tasks. More comprehensive details about YOLO are available in subsection 3.3.2.

#### 3.3.1 CountNet

CountNet (Bhattarai and Karkee, 2022) is a regression-based artificial neural network that does not require position annotations, making it conceptually similar to the method we propose. Consequently, it serves as the closest competitor for comparison in this study. We have implemented the method as closely as possible to its original paper description. The VGG16 network serves as the backbone, incorporating GAP at the output stage. The backbone is pretrained with Imagenet. The output of the backbone is then fed into a fully connected linear network. Dropout is implemented for generalization, and the ReLU function is used as the activation function. The Adam optimizer is paired with an exponential learning rate scheduler to enhance convergence during training. To achieve optimal results with the dataset provided, the only adjustments made were to the initial learning rate (1*e*−4) and learning rate decay (0.972351). The training lasts for 300 epochs, with the best epoch determined based on validation data.

#### 3.3.2 YOLO with linear regression

The implementation of YOLOv8n Ultralytics 8.0.225 is used as a reference method representing the conventional approaches based on segmentation or detection. The smallest variant of YOLO is selected due to the limited size of the dataset. The YOLOv8 (Jocher et al., 2023) is the eighth version of the You Only Look Once (YOLO) deep learning model. Version eight seems to be the most well-used version of YOLO currently, and simultaneously it is one of the most used models for both detection and segmentation. Therefore, it was used as a reference method. Furthermore, YOLO exhibits exceptional accuracy, consistently achieving state-of-the-art results on benchmark data sets for object detection and segmentation (Zou et al., 2023).

The YOLO model is trained with polygon annotations. As the YOLO model provides predictions indicating individual flower sets within an image, it is necessary to process both images of the tree to count all flower sets present. The subsequent total of flower sets derived from YOLO is refined using the following linear equation to enhance accuracy (Equation 5):

(5) $$y_{n,o}=a\left(y_{n,o,A}+y_{n,o,B}\right)+b,$$

where *y_n_*, *_o_* represents the adjusted flower set count, *y_n, o, s_*is the YOLO prediction for one side, and constants *a* and *b* are determined via linear regression to minimize error relative to the actual count. This correction compensates for the potential systematic overestimation or underestimation by YOLO, and it is commonly used for similar problems.

### 3.4 Hardware, software and implementation

All experiments with both models were performed on a computing node equipped with an AMD EPYC 9554 64-Core Processor (four CPU cores allocated), 1.5 TiB of RAM, NVMe storage, and an NVIDIA H100 GPU with 80 GB of HBM3 memory. The system ran Rocky Linux 9.6 (Blue Onyx) with NVIDIA driver 580.82.07. The code was written in Python 3.12.9 using PyTorch 2.9.1 with CUDA 12.8 support. All images and annotations were resized to 512x512 resolution. The batch size was adjusted to the highest possible number allowed by the memory capacity of the graphics card for all models used.

### 3.5 Evaluation metrics

The performance of flower cluster count estimations was assessed with the mean absolute error (MAE) (Equation 6), the root mean squared error (RMSE) (Equation 7), and the relative error (RE) (Equation 8). MAE and RMSE account for the estimated number of flower clusters in all trees. The RE is based on MAE and the mean flower count. Calculating RE per tree is not possible since certain trees have no flowers. The computation of these metrics is as follows:

(6) $$\text{MAE}=\frac{1}{NO}\sum_{n=1}^{N}\sum_{o=1}^{O}\left|y_{n,o}-t_{n,o}\right|,$$

(7) $$\text{RMSE}=\sqrt{\frac{1}{NO}\sum_{n=1}^{N}\sum_{o=1}^{O}{\left(y_{n,o}-t_{n,o}\right)}^{2}},$$

(8) $$\text{RE}=\frac{\text{MAE}}{\frac{1}{NO}\sum_{n=1}^{N}\sum_{o=1}^{O}t_{n,o}}\cdot 100\%,$$

where *N* is the total number of trees in the validation set, *O* is the total number of observations per tree, *y_n_*, *_o_* is the predicted count, and *t_n_*,*_o_* is the target count (field-validated or visually-estimated).

## 4 Experiments and result

As certain trees feature both a daytime and a nighttime observation, the dataset was divided by tree rather than by observation. While this results in an uneven quantity of images across the divisions, it effectively avoids any data leakage. This ensures that different images from the same tree do not simultaneously appear in both the training and validation datasets.

Due to the limited size of the dataset, the repeated 10-fold crossvalidation with 10 repeats was performed. This experiment structure ensures that all trees (not observations) were included in the validation set, while avoiding potential data leakage. Additionally, the experiment was conducted ten separate times with a different random seed to obtain robust results. In essence, the proposed model (and also the reference model) underwent training 100 times, as a result of multiplying 10 folds by 10 experiments.

### 4.1 Comparative performance study

The proposed method was compared with two reference methods: CountNet and YOLO detection-based counting. Because the CountNet and the proposed method do not work with exact polygon annotations, they are not used in comparison. All three methods were compared against field-validated counts and visually-estimated counts. The results are summarized in **Table 4**. As shown in the table, the proposed method outperforms both reference methods in both experiments. The MAE difference of the proposed method and the second-best method (CountNet) is 0.5 flower clusters per observation in field-validated counts, and a difference of 0.20 in visually-estimated counts.

**Table 4:** Evaluation of the methods based on MAE, RMSE, RE, and Pearson correlation, with top results emphasized in bold.

Field-validated counts				
Method	MAE	RMSE	RE	Correlation
Proposed method	**2.08** ± 0.15	**3.51**	**10.26%**	**0.972**
CountNet (Ref. 1)	2.58 ± 0.10	4.39	12.72%	0.956
YOLO (Ref. 2)	3.58 ± 0.14	5.08	17.61%	0.961
Visually-estimated counts				
Method	MAE	RMSE	RE	Correlation
Proposed method	**1.40** ± 0.07	**2.29**	**7.82%**	**0.984**
CountNet (Ref. 1)	1.60 ± 0.09	2.50	8.95%	0.980
YOLO (Ref. 2)	3.56 ± 0.10	4.56	19.91%	0.951

The proposed model comprises around 26.1 million parameters, whereas CountNet has 14.9 million and YOLO comprises 3.4 million parameters. Despite these differences, the proposed method’s average inference time is 14 milliseconds, compared to CountNet’s 21 milliseconds and YOLO’s 10 milliseconds. While a precise comparison of inference time and model complexity falls beyond this study’s scope—given the restriction to a single hardware and software configuration—these rudimentary results indicate significant disparities in inference speed, as expected.

### 4.2 Ablation study

Compared to CountNet, which utilizes VGG16, the proposed approach employing ResNet appears more promising for the specified challenge. However, integrating FPN with ResNet requires additional clarification, as adding FPN increases the model’s parameters from approximately 24.7M to 26.1M. Thus, we conducted an ablation study, contrasting the proposed method with an approach without FPN. As shown in **Table 5**, the findings clearly indicate that integrating FPN results in significant improvements to the proposed architecture. In addition, we investigate the use of exponential learning rate decay strategy, which is more common than the cosine annealing proposed in the main study of this paper. The experimentally chosen parameter of exponential decay is 0.95. As shown in **Table 5**, the cosine annealing seems to be a better alternative for the given experimental settings and data.

**Table 5:** Ablation study of the proposed method on field-validated counts.

FPN	LR decay	Fusion	MAE	RMSE	RE	Correlation
Yes	Annealing	Concat	**2.08** ± **0.15**	3.51	10.26%	0.972
Yes	Annealing	Sum	2.10 ± 0.12	**3.44**	10.36%	**0.973**
Yes	Annealing	Attention	2.15 ± 0.16	3.54	10.60%	0.971
No	Annealing	Sum	2.26 ± 0.14	3.71	11.14%	0.969
No	Annealing	Attention	2.28 ± 0.14	3.74	11.21%	0.968
No	Annealing	Concat	2.28 ± 0.10	3.76	11.24%	0.968
Yes	Exponential	Sum	2.36 ± 0.14	3.82	11.60%	0.967
Yes	Exponential	Concat	2.36 ± 0.17	3.78	11.64%	0.967
Yes	Exponential	Attention	2.40 ± 0.14	3.91	11.79%	0.965
No	Exponential	Concat	2.53 ± 0.13	4.00	12.45%	0.964
No	Exponential	Attention	2.56 ± 0.14	4.03	12.59%	0.963
No	Exponential	Sum	2.60 ± 0.11	4.19	12.80%	0.960

Results are sorted by MAE, from best to worst. Bold values indicate the best-performing configuration across all evaluated metrics.

Furthermore, we investigate different strategies for fusing information from the two tree views. The proposed method employs feature concatenation before the regression head. As alternatives, we evaluate *sum fusion*, where each view is processed independently and the final count is obtained and *attention fusion*, where the two feature vectors are treated as a two-token sequence and processed by a multi-head self-attention module before regression. This strategy allows information exchange between the two views and enables view-dependent feature weighting.

As shown in **Table 5**, cosine annealing consistently outperforms exponential learning rate decay across all tested configurations. The inclusion of FPN provides the second most significant improvement, yielding lower errors for both learning rate schedules and all fusion strategies. In contrast, the choice of fusion strategy has a relatively minor impact on performance. While the best MAE is achieved by feature concatenation, the differences between concatenation and summation are small and not consistent across all evaluation metrics, indicating that the overall architecture and optimization strategy are more influential than the specific fusion mechanism.

### 4.3 Importance of a multi-view input

The whole idea of dual-view (or multi-view) input is based on two assumptions:

- It is not possible to see all flower clusters of a tree in one image.
- Images of the same tree taken from different angles can contain different number of flower clusters.

For the dataset employed in this study, two images are captured from precisely opposing directions (sides A and B). Based on number of polygon annotations per side, the average discrepancy between sides A and B amounts to 2.52 flower clusters per tree, equating to 20.42% of the mean flower cluster count per side. The largest discrepancy observed within a single tree between the sides is 16 flower clusters, with a Pearson correlation coefficient of 0.91 for flower cluster counts on either side. This information illustrates that an image from just one side offers only a partial view, insufficient to accurately estimate the tree’s flower cluster count. Therefore, the proposed model combines feature representations extracted from both sides of the tree to leverage complementary observations.

## 5 DISCUSSION

While our study demonstrates the potential of a deep regression-based model for apple flower cluster counting, several limitations should be considered. First, the dataset was collected from one orchard, comprising only columnar apple trees from three specific varieties. Although the images were captured both during the day and at night, they were taken under limited weather conditions, which may restrict the dataset’s representativeness. The results indicate the efficacy of the proposed method for columnar apple trees captured from two angles; however, variations in tree structure may require an altered number of images.

Furthermore, the accuracy of flower cluster counting heavily depends on the quality of manual annotations, a challenging and intricate task that may introduce biases or errors into the dataset.

The phenological period of inflorescence can influence the counting accuracy, particularly if field validation and imaging are not conducted simultaneously. Flower clusters may change in appearance between the time of imaging and manual validation. These temporal differences can introduce discrepancies in the recorded flower count. To achieve the highest accuracy, field-validated counts should be obtained as close in time to imaging as possible.

Additionally, the dataset, though sufficient for training and validating the proposed method, is relatively small compared to those used in similar deep learning studies. A larger, more diverse dataset—or one explicitly designed for external validation—could further enhance the model’s performance and generalizability, particularly to analyze the impact of lighting and weather conditions during the imaging process.

**Figure 3:**
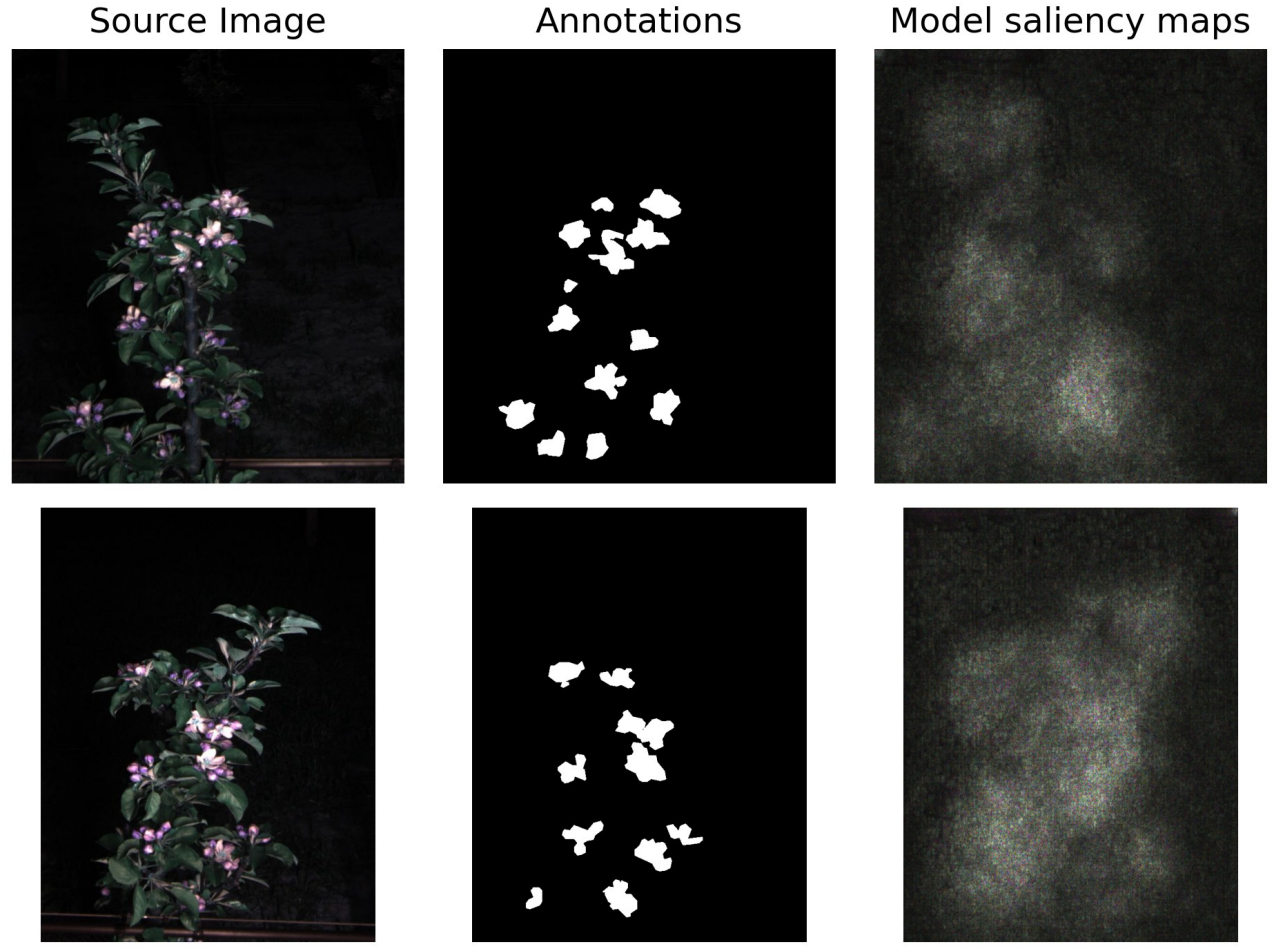
Examples of model inputs, annotations, and saliency maps. The images show both sides of a tree used as inputs to the model, corresponding human-generated flower cluster count annotations, and saliency maps computed from the gradients of the proposed model. The saliency maps highlight image regions that contribute most strongly to the predicted count.

One key factor contributing to the limited adoption of regression-based models is the difficulty of interpreting their predictions. Unlike detection-based approaches, regression models provide counts without explicit object localization. **Figure 3** presents representative saliency maps indicating that the model predominantly focuses on regions corresponding to the tree structure and flower clusters. Importantly, activation extending beyond the flowers themselves should not necessarily be interpreted negatively, as leaves and branches provide valuable contextual information regarding flower distribution and density.

Therefore, the observed saliency patterns suggest that the model exploits meaningful plant-related features rather than unrelated image artifacts or data leakage.

## 6 Conclusion

This research introduces a deep regression-based method to estimate the flower cluster count of apple trees through image analysis. Key findings from this study are as follows:

- The object detection-based counting methodologies are significantly influenced by data annotation, showing a notable difference of 29.53% in the label counts within the dataset examined.
- Annotations using boxes or polygons, intended for detection or segmentation, fail to accurately capture the true number of flower clusters present in an actual tree.
- Regression-based methods have demonstrated superior performance compared to conventional detection-based model in our experiments.
- This paper proposes a potentially superior regression alternative to CountNet in terms of regression performance and inference time.
- It is essential to use multiple images of a tree to achieve accurate estimate of flower cluster counts possible.
- Moreover, CountNet has been validated on a new dataset with our extensive validation setup (10 times repeated 10-fold cross-validation).

The above conclusions suggest that transitioning from commonly used detection-based to less utilized regression-based models could greatly enhance counting precision in agriculture and decrease the need for precisely located annotations.

## Data Availability

The datasets presented in this study can be found in online repositories. The names of the repository/repositories and accession number(s) can be found in the article/supplementary material.

## References

[B1] Achanta R. Shaji A. Smith K. Lucchi A. Fua P. Süsstrunk S. (2012). Slic superpixels compared to state-of-the-art superpixel methods. IEEE Trans. Pattern Anal. Mach. Intell. 34, 2274–2282. doi: 10.1109/tpami.2012.120 22641706

[B2] Altaheri H. Alsulaiman M. Muhammad G. (2019). Date fruit classification for robotic harvesting in a natural environment using deep learning. IEEE Access 7, 117115–117133. doi: 10.1109/access.2019.2936536 25079929

[B3] Badrinarayanan V. Kendall A. Cipolla R. (2017). Segnet: A deep convolutional encoder-decoder architecture for image segmentation. IEEE Trans. Pattern Anal. Mach. Intell. 39, 2481–2495. doi: 10.1109/tpami.2016.2644615 28060704

[B4] Bhattarai U. Bhusal S. Zhang Q. Karkee M. (2024). Agregnet: A deep regression network for flower and fruit density estimation, localization, and counting in orchards. Comput. Electron. Agric. 227, 109534. doi: 10.1016/j.compag.2024.109534 38826717

[B5] Bhattarai U. Karkee M. (2022). A weakly-supervised approach for flower/fruit counting in apple orchards. Comput. Ind. 138, 103635. doi: 10.1016/j.compind.2022.103635 38826717

[B6] Bochkovskiy A. Wang C.-Y. Liao H.-Y. M. (2020). Yolov4: Optimal speed and accuracy of object detection. arXiv preprint, arXiv:2004.10934. doi: 10.48550/arXiv.2004.10934

[B7] Bound S. A. (2018). Precision crop load management of apple (malus x domestica borkh.) without chemicals. Horticulturae 5, 3. doi: 10.3390/horticulturae5010003 30654563

[B8] Cai Y. Zhang P. Wang X. (2024). A weakly supervised crowd counting method via joint cnn and transformer network. Electronics 13, 5053. doi: 10.3390/electronics13245053 30654563

[B9] Campbell T. Kalcsits L. (2024). Strategies to overcome biennial bearing in apple – a review. Eur. J. Agron. 158, 127213. doi: 10.1016/j.eja.2024.127213 38826717

[B10] Chen C.-J. Huang Y.-Y. Li Y.-S. Chen Y.-C. Chang C.-Y. Huang Y.-M. (2021). Identification of fruit tree pests with deep learning on embedded drone to achieve accurate pesticide spraying. IEEE Access 9, 21986–21997. doi: 10.1109/access.2021.3056082 25079929

[B11] Ciregan D. Meier U. Schmidhuber J. (2012). Multi-column deep neural networks for image classification. In: 2012 IEEE Conference on Computer Vision and Pattern Recognition (CVPR) (Providence, RI: IEEE), 3642–3649.

[B12] Deng J. Dong W. Socher R. Li L.-J. Li K. Fei-Fei L. (2009). Imagenet: A large-scale hierarchical image database. In: 2009 IEEE Conference on Computer Vision and Pattern Recognition (CVPR) (Miami, FL: IEEE), 248–255.

[B13] Dias P. A. Tabb A. Medeiros H. (2018). Apple flower detection using deep convolutional networks. Comput. Ind. 99, 17–28. doi: 10.1016/j.compind.2018.03.010 38826717

[B14] Estrada J. S. Vasconez J. P. Fu L. Cheein F. A. (2024). Deep learning based flower detection and counting in highly populated images: A peach grove case study. J. Agric. Food Res. 15, 100930. doi: 10.1016/j.jafr.2023.100930 38826717

[B15] Farjon G. Krikeb O. Hillel A. B. Alchanatis V. (2020). Detection and counting of flowers on apple trees for better chemical thinning decisions. Precis. Agric. 21, 503–521. doi: 10.1007/s11119-019-09679-1 30311153

[B16] Gomez A. S. Aptoula E. Parsons S. Bosilj P. (2021). Deep regression versus detection for counting in robotic phenotyping. IEEE Rob. Autom. Lett. 6, 2902–2907. doi: 10.1109/lra.2021.3062586 25079929

[B17] He K. Zhang X. Ren S. Sun J. (2016). Deep residual learning for image recognition. In: Proceedings of the IEEE Conference on Computer Vision and Pattern Recognition (CVPR 2016) (Las Vegas, NV: IEEE), 770–778. doi: 10.1109/cvpr.2016.90

[B18] He K. Gkioxari G. Dollár P. Girshick R. (2017). Mask r-cnn. In: Proceedings of the IEEE International Conference on Computer Vision (ICCV 2017) (Venice: IEEE), 2961–2969.

[B19] He L. Fang W. Zhao G. Wu Z. Fu L. Li R. . (2022). Fruit yield prediction and estimation in orchards: A state-of-the-art comprehensive review for both direct and indirect methods. Comput. Electron. Agric. 195, 106812. doi: 10.1016/j.compag.2022.106812 38826717

[B20] Hu J. Fan C. Wang Z. Ruan J. Wu S. (2023). Fruit detection and counting in apple orchards based on improved yolov7 and multi-object tracking methods. Sensors 23, 5903. doi: 10.3390/s23135903 37447752 PMC10347148

[B21] Jocher G. (2020). Software repository. ( Ultralytics). doi: 10.5281/zenodo.3908559

[B22] Jocher G. Chaurasia A. Qiu J. (2023). Software repository. ( Ultralytics). Available online at: https://github.com/ultralytics/ultralytics.

[B23] Li G. Suo R. Zhao G. Gao C. Fu L. Shi F. . (2022). Real-time detection of kiwifruit flower and bud simultaneously in orchard using yolov4 for robotic pollination. Comput. Electron. Agric. 193, 106641. doi: 10.1016/j.compag.2021.106641 38826717

[B24] Lin T.-Y. Dollár P. Girshick R. He K. Hariharan B. Belongie S. (2017). Feature pyramid networks for object detection. In: Proceedings of the IEEE Conference on Computer Vision and Pattern Recognition (CVPR 2017) (Honolulu, HI: IEEE), 2117–2125.

[B25] Lin J. Li J. Ma Z. Li C. Huang G. Lu H. (2024). A framework for single-panicle litchi flower counting by regression with multitask learning. Plant Phenomics 6, 172. doi: 10.34133/plantphenomics.0172 38629081 PMC11018488

[B26] Lin J. Li J. Yang Z. Lu H. Ding Y. Cui H. (2022). Estimating litchi flower number using a multicolumn convolutional neural network based on a density map. Precis. Agric. 23, 1226–1247. doi: 10.1007/s11119-022-09882-7 30311153

[B27] Lin T.-Y. Maire M. Belongie S. Bourdev L. Girshick R. Hays J. (2015). Microsoft coco: Common objects in context. In: Computer Vision – ECCV 2014, Lecture Notes in Computer Science, Vol. 8693 (Cham, Switzerland: Springer), 740–755. doi: 10.1007/978-3-319-10602-1_48

[B28] Loshchilov I. Hutter F. (2017). Fixing weight decay regularization in adam. arXiv preprint, arXiv:1711.05101. doi: 10.48550/arXiv.1711.05101

[B29] Mann H. M. Iosifidis A. Jepsen J. U. Welker J. M. Loonen M. J. Høye T. T. (2022). Automatic flower detection and phenology monitoring using time-lapse cameras and deep learning. Remote Sens. Ecol. Conserv. 8, 765–777. doi: 10.1002/rse2.275 41531421

[B30] Mao J. Zhang Y. Liu Z. (2025). Learning to count crowds from low-altitude aerial views using weakly supervised feature-adaptive fusion. Appl. Sci. 15, 13211. doi: 10.3390/app152413211 30654563

[B31] Meier U. Bleiholder H. Buhr L. Feller C. Heß M. Lancashire P. . (2009). The bbch system to coding the phenological growth stages of plants-history and publications. J. Kulturpflanz. 61, 41–52. doi: 10.5073/JfK.2009.02.01

[B32] Meng H. Hong X. Lai Z. Shang M. (2025). Free lunch enhancements for multi-modal crowd counting. In: Proceedings of the IEEE/CVF Conference on Computer Vision and Pattern Recognition (CVPR 2025) (Nashville, TN: IEEE), 14013–14023.

[B33] Mo H. Zhang X. Tan J. Yang C. Gu Q. Hang B. . (2024). Countformer: Multi-view crowd counting transformer. In: Computer Vision – ECCV 2024, Lecture Notes in Computer Science, Part LII (Cham: Springer).

[B34] Mu X. He L. Heinemann P. Schupp J. Karkee M. (2023). Mask r-cnn based apple flower detection and king flower identification for precision pollination. Smart Agric. Technol. 4, 100151. doi: 10.1016/j.atech.2022.100151 38826717

[B35] Palacios F. Bueno G. Salido J. Diago M. P. Hernández I. Tardaguila J. (2020). Automated grapevine flower detection and quantification method based on computer vision and deep learning from on-the-go imaging using a mobile sensing platform under field conditions. Comput. Electron. Agric. 178, 105796. doi: 10.1016/j.compag.2020.105796 38826717

[B36] Rea R. Eccel E. (2006). Phenological models for blooming of apple in a mountainous region. Int. J. Biometeorol. 51, 1–16. doi: 10.1007/s00484-006-0043-x 16909259

[B37] Redmon J. Farhadi A. (2018). Yolov3: An incremental improvement. arXiv preprint, arXiv:1804.02767. doi: 10.48550/arXiv.1804.02767

[B38] Robinson T. Gonzalez L. Cheng L. Ziang Y. Peck G. Arnoldussen B. . (2022). Studies in precision crop load management of apple. In: XXXI International Horticultural Congress (IHC2022): International Symposium on Innovative Perennial Crops Management, Acta Horticulturae 1366 (Angers: ISHS (International Society for Horticultural Science)), 219–226.

[B39] Robinson T. Lakso A. Greene D. Hoying S. (2013). Precision crop load management. NY Fruit Q. 21, 6–9. doi: 10.17660/actahortic.2020.1281.53

[B40] Seguí S. Pujol O. Vitria J. (2015). Learning to count with deep object features. In: Proceedings of the IEEE Conference on Computer Vision and Pattern Recognition Workshops (CVPRW 2015) (Boston, MA: IEEE), 90–96.

[B41] Shang Y. Geng M. Fang H. Cai M. Wang J. Song H. (2024). Using unmanned aerial vehicle acquired RGB images and Density-Cluster-Count model for tree-level apple flower quantification. Comput. Electron. Agric. 226, 109389. doi: 10.1016/j.compag.2024.109389 38826717

[B42] Shorten C. Khoshgoftaar T. M. (2019). A survey on image data augmentation for deep learning. J. Big Data 6, 1–48. doi: 10.1186/s40537-019-0197-0 34306963 PMC8287113

[B43] Simonyan K. Zisserman A. (2014). Very deep convolutional networks for large-scale image recognition. arXiv preprint, arXiv:1409.1556. doi: 10.48550/arXiv.1409.1556

[B44] Tan C. Sun J. Paterson A. H. Song H. Li C. (2024). Three-view cotton flower counting through multi-object tracking and rgb-d imagery. Biosyst. Eng. 246, 233–247. doi: 10.1016/j.biosystemseng.2024.08.010 38826717

[B45] Tian H. Wang T. Liu Y. Qiao X. Li Y. (2020). Computer vision technology in agricultural automation—a review. Inf. Process. Agric. 7, 1–19. doi: 10.1007/978-981-10-1509-0_10 28220984

[B46] Verma P. Sharma S. Sharma N. Chauhan N. (2023). Review on crop load management in apple (malus x domestica borkh.). J. Hortic. Sci. Biotechnol. 98, 299–321. doi: 10.1080/14620316.2022.2149425

[B47] Wang C.-Y. Bochkovskiy A. Liao H.-Y. M. (2023). Yolov7: Trainable bag-of-freebies sets new state-of-the-art for real-time object detectors. In: 2023 IEEE/CVF Conference on Computer Vision and Pattern Recognition (CVPR) (Vancouver, BC: IEEE), 7464–7475.

[B48] Wang D. Song H. Wang B. (2025). YO-AFD: an improved YOLOv8-based deep learning approach for rapid and accurate apple flower detection. Front. Plant Sci. 16, 1541266. doi: 10.3389/fpls.2025.1541266 40144752 PMC11936985

[B49] Wertheim S. J. Webster A. D. (2005). Fundamentals of Temperate Zone Tree Fruit Production. (Leiden: Backhuys Publishers).

[B50] Xia X. Chai X. Li Z. Zhang N. Sun T. (2023). MTYOLOX: Multi-transformers-enabled YOLO for tree-level apple inflorescences detection and density mapping. Comput. Electron. Agric. 209, 107803. doi: 10.1016/j.compag.2023.107803 38826717

[B51] Yang X. Gao Y. Yin M. Li H. (2024). Automatic apple detection and counting with AD-YOLO and MR-SORT. Sensors 24, 7012. doi: 10.3390/s24217012 39517909 PMC11548465

[B52] Zahid A. Mahmud M. S. He L. Heinemann P. Choi D. Schupp J. (2021). Technological advancements towards developing a robotic pruner for apple trees: A review. Comput. Electron. Agric. 189, 106383. doi: 10.1016/j.compag.2021.106383 38826717

[B53] Zheng C. Liu T. Abd-Elrahman A. Whitaker V. M. Wilkinson B. (2023). Object-detection from multi-view remote sensing images: A case study of fruit and flower detection and counting on a central florida strawberry farm. Int. J. Appl. Earth Obs. Geoinf. 123, 103457. doi: 10.1016/j.jag.2023.103457 38826717

[B54] Zou Z. Chen K. Shi Z. Guo Y. Ye J. (2023). Object detection in 20 years: A survey. Proc. IEEE 111, 257–276. doi: 10.1109/jproc.2023.3238524 25079929

